# Leveraging family and caregiver support in psychedelic-assisted therapy: considerations for the treatment of adolescents

**DOI:** 10.1186/s13034-025-00930-4

**Published:** 2025-06-04

**Authors:** Sarah Forsberg, Amanda E. Downey, Lindsey D. Bruett, Lisa Hail, Gisele Fernandes-Osterhold, Josh D. Woolley, Adele Lafrance, Marissa Raymond-Flesch

**Affiliations:** 1 Department of Psychiatry and Behavioral Sciences, University of California, San Francisco, San Francisco, CA USA; 2 Department of Pediatrics, University of California, San Francisco, San Francisco, CA USA; 3 San Francisco Veteran’s Affairs Medical Center, San Francisco, CA USA; 4 Emotion Science, Department of Research, Denver, CO USA; 5 Philip R. Lee Institute for Health Policy Studies, University of California, San Francisco, CA USA

**Keywords:** Adolescence, Psychedelics, Family

## Abstract

**Background:**

Psychedelic-assisted therapy (PAT) is gaining recognition as a promising intervention for treatment-refractory mental health conditions in adults. While interest in its applications for adolescents is growing, critical gaps remain in understanding safe and effective delivery of PAT to this vulnerable population, including implications for caregiver/family (used interchangeably) involvement in the intervention. Caregivers play a crucial role in adolescent mental health treatment by supporting recovery and disrupting illness-maintaining patterns. They also benefit from resources to navigate the challenges of caregiving. This review synthesizes existing literature on evidence-based adolescent mental health treatments, best practices in PAT delivery, and family systems theory to propose a framework for integrating caregivers in adolescent PAT.

**Conclusion:**

Current literature in both PAT and adolescent mental health provides a compelling case for the direct involvement of caregivers in PAT for adolescent populations. In modern PAT, therapeutic setting (i.e., “set and setting”) is increasingly recognized as essential for optimizing outcomes. Similarly, the family system serves as a critical setting that may shape PAT efficacy in adolescents. This review provides recommendations and unique considerations for the incorporation of caregivers into PAT protocols for adolescents to maximize safety and efficacy of the intervention.

## Background

Adolescence, broadly defined as the transitional stage between childhood and adulthood, is the period during which up to three-quarters of all lifetime mental health conditions first manifest [1, 2]. This developmental period presents the opportunity for maturation of emotion regulation and executive function, while also increasing vulnerability to psychopathology [3, 4]. The increasing prevalence of mental health problems, including suicide, prompted the declaration of a National Health Emergency in Child and Adolescent Mental Health by the American Academy of Pediatrics, American Academy of Child and Adolescent Psychiatry and Children’s Hospital Association following the COVID-19 pandemic [5]. Addressing the adolescent mental health crisis will require diverse approaches and substantial resources to increase access to existing evidence-based treatments (EBTs) and expand novel treatments for those who remain unresponsive.

Given the significant mental health crisis facing adolescents and the limited efficacy and accessibility of EBTs, there is growing interest in adapting psychedelic-assisted therapy (PAT) for adolescents [6, 7]. PAT shows promise in treating adults with mental health conditions characterized by chronicity, functional limitation, and limited response to conventional treatments [8–11]. Modern PAT precedes through three defined stages: preparation, dosing, and integration [12–14]. First, preparation sessions allow the therapist and patient to establish a therapeutic alliance. In the context of this relationship, the therapist reviews the logistics for drug administration, explains safety considerations, assists the participant in establishing intentions, and establishes boundaries to support safely navigating challenges that may arise during dosing. On the day of drug administration (“dosing day”), the therapist(s) is present with the patient for the duration of acute drug effects, monitoring physiological safety and providing psychological support. Most modern clinical trials of PAT incorporate one or two high-dose psychedelic drug administration sessions. Finally, in integration sessions, the therapist(s) works with the patient to explore content that arose during drug administration, making meaning of the experience and incorporating insights into daily life [13].

With promising evidence in adults, adapting PAT for adolescents requires careful consideration of stage-specific developmental trajectories [15, 16]. Adolescence is a period of profound neurodevelopmental change, marked by increasing capacities for abstract thought, emotional regulation, and logical reasoning, but also heightened sensitivity to social influences [1]. And while complex cognitive processing abilities rapidly expand during adolescence, continued cognitive refinement continues through the third decade of life, suggesting that decision making processes, risk assessment, and the integration of novel experiences– including those induced by psychedelics– may differ substantially between adolescents and fully matured adults. While clinical trial data in adolescents are lacking, a naturalistic study of recreational use in 16-24-year-olds suggested that psychedelic induced ego-dissolution may be associated with lower psychological well-being, opposite to adult findings [16]. Adolescents also reported more challenging experiences and visual abnormalities resembling hallucinogen persisting perceptual disorder [16]. Though not statistically significant and limited by study design, these findings signal potential vulnerabilities for adolescents undergoing PAT. Given these risks and the inherent vulnerability posed by adolescent neurodevelopment, a developmentally informed PAT model is warranted.

Given the many unknowns about the potential risks and benefits of PAT in adolescents with mental health conditions, if and how PAT should be delivered to adolescents remains uncertain. Unlike in adults who have reached neural maturation, adolescents are increasingly vulnerable to rapid neural changes contributing to dysregulation and may not have fully developed executive functioning to meet these challenges. Mental health challenges may even reflect insufficient psychological stability rather than excessive rigidity, raising important questions about the appropriateness of interventions that further increase neural plasticity; however, a full examination of these developmental considerations lies beyond the scope of this paper. In this review, we focus on the incorporation of caregivers and families (used interchangeably to encompass the caregiving/home environment of the adolescent) directly into the intervention as one potential approach to not only mitigate risks but also enhance the possibility of positive, durable change. Parents and caregivers may also serve as a developmentally appropriate external source of stability during this period, further highlighting the utility of their involvement. First, we review the evidence supporting caregiver involvement in adolescent mental health treatment broadly. Second, we discuss the growing field of PAT and explore developmental considerations for the use of PAT in adolescents. Finally, we propose a preliminary roadmap for the inclusion of caregivers in PAT for adolescents, specifically in assessments of developmental readiness, informed consent and assent, and the intervention itself.

### Caregiver involvement in evidence-based treatments (EBTs) for youth

Historically, families were often viewed as sources of dysfunction, with adolescent mental health treatment thought to require separation from the family [17, 18]. We take the stance that attributing mental illness to family or parenting is reductionistic and has historically led to harm. Mental health disorders result from the complex interplay between genetics and environment across development [19]. Therefore, attention in mental health treatment to biology and environment [20], the transmission of intergenerational trauma [21], and the ways family systems may be bi-directionally affected by the presence of mental illness [22] is warranted. A shift towards family-engaged approaches may mitigate the well-established psychological, social, physical and economic strain on caregivers who support a child with mental illness through reducing blame and isolation and increasing resources for caregivers [23, 24]. Family-engaged approaches also acknowledge the significant influence of social determinants of mental health (e.g. structural racism, inequitable access to health care, education, stigma associated with mental illness) that impact the whole family system [25, 26]. Further, caregivers play a central role in providing logistical support to adolescents navigating the healthcare system, be it for managing chronic illnesses or receiving regular primary care. In this fundamental role, integrating caregivers into PAT aligns with standard approaches in psychiatric, pediatric and adolescent medicine broadly, ensuring continuity and reinforcing a supportive environment for care. Together, family-engaged approaches are increasingly shown to enhance the efficacy of interventions for adolescents, even among interventions originally designed for individual delivery [27–30].

While the extent of caregiver engagement is model-dependent, core principles spanning modalities often include: (1) psychoeducation to enhance caregiver understanding of their adolescent’s mental health; (2) education about the treatment approach to help caregivers reinforce treatment-consistent behavior and avoid counterproductive interactions (e.g., accommodating anxiety); and (3) direct caregiver skill-building to support implementation of effective behavioral strategies at home (e.g., meal support for eating disorders, exposure support for anxiety disorders) and to improve communication strategies (e.g., validation to support youth emotion regulation, contingency management to support substance use reduction). Across diagnostic groups, many EBTs outline structured caregiver interventions, whether they be caregiver-only or conjoint sessions with the adolescent [31–37]. EBTs for adolescents also include transdiagnostic family models originating from attachment theory, which posits that the caregiver-child attachment is foundational for mental health [38]. They have further evolved to emphasize the equal importance of addressing caregiver emotional needs—such as intergenerational attachments, distressing experiences, and caregiving strain—to support the broader system [27], with models like Emotion-Focused Family Therapy (EFFT) focusing on addressing caregiver needs as well [35, 36, 38].

Across these models, caregiver-inclusive treatment protocols aim to help families foster environments that support lasting behavioral and emotional change for their adolescents. This approach may be particularly well-suited to PAT, as it equips the family system to prepare for and modify their responses to positive change resulting from a psychedelic experience. Given the growing evidence supporting caregiver involvement in adolescent mental health interventions, integrating this framework into PAT for adolescents may both enhance therapeutic outcomes and mitigate potential risks.

### Psychedelic-assisted psychotherapy and adolescence

In some Indigenous communities, youth engage in ceremonial use of psychedelic substances, contingent on cultural views of developmental readiness [39]. Classic psychedelics like psilocybin and lysergic acid diethylamide are serotonin receptor (5-HT2A) agonists hypothesized to acutely enhance cognitive flexibility [40, 41], potentiate a hyperplastic neurobiological state [42, 43], and relax prior beliefs [44–46]. The neurobiological mechanisms underlying both the acute and long-lasting effects of PAT, as well as the contribution of psychotherapy before, during, and after the drug administration session(s), remain areas of active scientific inquiry. However, acute effects of these psychoactive substances include the dysregulation of neural circuitry, possibly allowing the opportunity for cognitive reorganization and new patterns of thought and behavior under the conditions of enhanced neural plasticity [43, 44, 47].

Together with the neurobiological changes described above, therapeutic efficacy of PAT is also proposed to depend on the “set” (i.e., mindset, unique aspects of the individual– their personality, preparation, intentions, and expectations) and “setting” (the physical, social, and cultural context) in which PAT occurs [48]. A third factor, “The Matrix,” hypothesizes that the impact of the broader sociocultural environment should be considered both before and following the psychedelic experience [49]. These contextual domains align with Bronfenbrenner’s Ecological Systems Theory, in which the adolescent’s experience is shaped by nested layers of influence—from the individual and interpersonal (e.g., caregiver involvement, peer support) to broader cultural and societal factors [50]. This framework can help identify and incorporate developmentally and culturally relevant variables that may impact engagement with, and response to, PAT. While “set” and “setting” are commonly considered in contemporary research, most modern clinical trials of PAT pay limited attention to the individual’s post-treatment environment (i.e., family, home life, resources, access to healthcare). Indeed, adolescents have limited autonomy in the context of the family system, e.g., where they live and with whom, which may heighten difficulties in enacting positive change to result from PAT [51]. Without adequate support in the home environment (e.g., teaching caregivers to validate their youth’s emotional experiences), returning adolescents to the same “matrix” in which symptoms developed may compromise treatment efficacy [51].

Thoughtful caregiver involvement in preparation and integration may leverage interpersonal connections, fostering a healthier adolescent sense of self in relationship [52]. Incorporating caregivers in preparation and integration may also help to increase caregiver responses that support the therapeutic process and positive behavioral change, while helping to minimize disruptions to the family system. Given the potential for psychedelics to enhance neuroplasticity, an engaged caregiver system may play a critical role in shaping new, adaptive neural pathways and reinforcing durable positive change within the adolescent’s home environment [51].

We propose engaging caregivers in PAT protocols for adolescents, incorporating what is known from existing adolescent-focused EBTs and psychedelic treatment models, as well as from our research program’s experience involving caregivers in preparation and integration as part of a clinical trial of psilocybin therapy for young adults with anorexia nervosa, illustrated in greater detail below (NCT06399263). Recent work has outlined key developmental and ethical considerations for MDMA-assisted psychotherapy in adolescents, emphasizing the importance of tailored adaptations, including caregiver involvement, and rigorous clinical evaluation before broader implementation [53]. While our recommendations may have relevance across adolescence, they are likely most applicable to older adolescents (ages 16+), given the developmental capacities required for participation in PAT and the age range reflected in the majority of studies cited. We provide a theoretical roadmap to guide researchers and clinicians in (1) informing assessments and readiness for the intervention, (2) tailoring consent and assent for caregivers and adolescents, and (3) effectively involving caregivers in the preparation and integration sessions of PAT. We also highlight outstanding challenges and questions of this approach.

### Leveraging caregivers to provide better assessments of individual-level risks and developmental readiness for PAT

*Recommendation*: Assessment of adolescent mental health involves gathering data from multiple informants to enhance conceptualization of treatment targets [54, 55]. For example, caregivers have a unique lens into the adolescent’s developmental trajectory, including exposure to developmental stressors, psychological and social development, capacity for emotion regulation and abstract reasoning, and the ability to process metaphorical or symbolic experiences, which may be particularly relevant given the nature of psychedelic experiences. Caregiver assessments can enrich awareness of potential challenges, resiliencies, and treatment targets that may impact PAT.

*Challenges and outstanding questions*: Caregivers and adolescents may express different views of symptoms, their impact, and related treatment goals. Synthesizing conflicting perspectives is a unique challenge of this developmental period, as reports can be informed by several factors. For example, adolescence is a time associated with a normative desire for autonomy and privacy, and perspective on mental health may be influenced by insight, social norms, perceived degree of distress, fear of potential negative outcomes if symptoms are disclosed, and patterns of denial and minimization. Integrating multiple perspectives often requires a sophisticated understanding of how to triangulate conflicting reports and maintain appropriate confidentiality for the adolescent. While state protections regarding confidentiality for adolescent mental health care vary, best practice holds that both adolescents and their caregivers should be informed about confidentiality and known limitations to confidentiality laws (e.g., threats of harm to self or others) that may arise during an intervention [56, 57]. Arriving at consensus on best practices in the assessment of baseline characteristics and developmental readiness prior to PAT will require collaboration between experts, caregivers, and adolescents.

### Assessing caregiver/family readiness for PAT

*Recommendation*: Caregiver resources and barriers should also be assessed prior to PAT and bolstered where possible. This may include social determinants of mental health, interpersonal factors like attachment history, capacity for emotion regulation within the family, exposure to relational trauma between the adolescent and their caregiver(s), and prior experiences in psychotherapy (for adolescent or their caregiver) that may impact expectations for the intervention. Similarly, ongoing psychotherapy and medical support after PAT requires financial and logistical support, which may be contingent on caregivers’ ability to secure insurance coverage and coordinate care. These practical considerations reinforce the importance of caregiver involvement in PAT, not only for emotional and psychological support, but also to ensure continuity of care and access to essential resources. Assessment should seek to avoid biases that privilege the most “capable” or “workable” caregiver(s)-—such as those with more time, internal or external resources, or cultural or linguistic congruence with providers—so as not to inadvertently exclude or marginalize families with fewer resources who may be equally in need of support.

*Challenges and outstanding questions*: Working with family systems may be a novel area for many clinicians trained in PAT, and even for some adolescent-focused clinicians. There are currently no standardized training requirements for PAT clinicians, nor standardized and evidence-based methodologies for incorporating caregivers into the intervention, leaving a critical gap in ensuring clinician competency. Establishing clear guidelines on the modality, intensity, and scope of training—particularly around developmental considerations, caregiver involvement, and risk mitigation for adolescents—will be needed. Additionally, the efficient yet comprehensive assessment of caregiver history and resources would benefit from standardization. These frameworks should evaluate readiness while ensuring equity across different family structures and backgrounds. In sum, best practices are needed to guide clinician training, caregiver involvement, and equitable assessment protocols to ensure safe, developmentally appropriate, and effective delivery of PAT within diverse family systems.

*Challenges and outstanding questions*: Identifying unresolved or active abuse between the caregiver and adolescent is critical before proceeding with PAT. A history of unresolved or current abuse between the caregiver and adolescent may be a contraindication, as psychedelic experiences may amplify trauma-related content that cannot be safely processed in their current environment [58, 59]. Clinicians will need a structured framework to assess both the presence of ongoing abuse and the lingering impact of past trauma on the caregiver-adolescent relationship. Determining what constitutes “active” abuse can be complex—this may refer only to recent incidents, but may also encompass a pattern of coercion, neglect, or relational harm that may still shape family dynamics. While ongoing abuse is likely a contraindication, situations where abuse has ceased but repair is lacking present a gray area that requires advanced skills on behalf of the clinician to ascertain appropriateness to move forward with the intervention. Fostering repair, reconnection, and a sense of emotional safety between caregivers and the adolescent may serve as a key therapeutic target for PAT.

### The informed consent and assent process

*Recommendation*: Prior work within adolescent psychopharmacology and psychotherapy consent emphasizes the importance of tailoring informed consent and assent procedures to developmental stage, assessing capacity in context, and carefully weighing therapeutic uncertainty against potential future benefit to others [60, 61]. To facilitate informed assent, adolescents should receive clear, developmentally appropriate explanations of the psychedelic compound, research protocols, and the potential benefits, risks, and uncertainties of the experience. The assent process should be tailored to the adolescent’s cognitive and emotional development, ensuring they are actively engaged in decision-making to the best of their ability [61]. Adolescents should be able to articulate motivators for participation that are self-driven and aligned with the goals of the intervention, thus providing informed assent prior to the intervention. In parallel, caregivers need to provide informed consent prior to the intervention. Adolescents and caregivers should participate in separate discussions in addition to a joint conversation, ensuring that each can express their perspectives independently [7]. Additionally, ongoing communication with the participant throughout the trial is essential, particularly in multi-dose studies, to confirm that adolescents continue to provide informed, voluntary assent as their understanding and perspectives evolve. It is important to note that some challenges in informed decision-making and consent/assent for PAT are not entirely unique to adolescents, as adults may also struggle to grasp the implications of participating in a psychedelic drug administration session with unique and highly variable experiences [6, 62]. Indeed, some argue that arbitrarily excluding adolescents under 18 who are otherwise capable decision-makers constitutes unjust age-based discrimination, particularly when strong informed consent/assent processes are in place [63].

*Challenges and outstanding questions*: Obtaining informed assent from adolescents in PAT presents several challenges, particularly given the unique, highly variable, and unpredictable nature of psychedelic experiences. A central question is how to design an assent and consent process that honors adolescent autonomy while ensuring appropriate caregiver involvement. Although separate conversations can help clarify the motivations and concerns of both adolescents and caregivers, questions remain about the degree to which adolescents can fully comprehend the possible effects of psychedelic experiences. Some argue that adolescents 16 and older generally possess the cognitive capacity and developmental readiness to provide informed assent to PAT [6]. However, in multi-dose studies, the adolescent’s perspectives may evolve as they gain experience with the treatment, particularly if the first experience is challenging or transformative. This raises important questions about how to assess ongoing voluntary assent throughout the course of a study. It is important to recognize that adolescents participating in PAT research must navigate layered consent—not only to a novel intervention but also to an investigational clinical trial. This dual role introduces additional ethical complexity, particularly when the personal therapeutic benefit is uncertain, and participation may primarily serve the advancement of knowledge for future patients. Transparency about this distinction should be a central feature of the informed consent and assent process. In sum, the assent process for adolescents in PAT trials requires a developmentally sensitive and iterative approach—yet standardized frameworks to guide this process are lacking.

### Caregiver involvement in preparation and integration

*Recommendation*: We advocate for the inclusion of caregivers in preparation and integration. The role, extent, and structure of caregiver involvement will require consideration of the therapeutic frame and clinical population. For example, caregivers may have sessions alone or together with the adolescent, or a combination of the two. In the context of heightened expressed emotion, for example (i.e. criticism and hostility), caregivers may benefit from separate sessions alone with the psychedelic therapist, mirroring the preparation and integration phases of PAT, as data suggests that separated support may enhance adolescent outcomes when expressed emotion is high [64, 65]. The possibility of caregiver-only or conjoint sessions warrants future exploration. We do not recommend for or against such approaches here but note that they raise distinct ethical and developmental questions that would benefit from empirical research. During the preparation phase, family cultural practices may be assessed and subsequently explored (prior beliefs about psychedelics, religious or spiritual practices). Existing family spiritual and cultural practices or belief systems may serve as a reference point during integration sessions to facilitate exploration of new insights, perspectives or mystical and spiritual experiences in support of meaning-making and behavior change. Preparing caregivers with tools that may buffer against some of the impacts on the family system (both positive and challenging) and reinforce positive behavioral adaptations in the home environment is presumed important to maximize the therapeutic benefit of PAT [51, 66].

*Challenges and outstanding questions*: While caregiver participation in preparation and integration may enhance the adolescent’s ability to process and sustain therapeutic insights, the degree of involvement necessary to achieve these benefits remains unknown. A key challenge lies in structuring caregiver participation in ways that are developmentally appropriate and culturally sensitive, ensuring that involvement fosters support rather than inadvertently reinforcing existing patterns of dysfunction. Further, while integrating family cultural practices into PAT may promote meaning-making, not all families have established traditions that could be incorporated. Similarly, caregiver psychoeducation and direct involvement are likely beneficial, but the optimal structure, duration, and content of these interventions remain unclear. Moreover, preparing caregivers with tools to buffer against challenging experiences—such as increased emotional vulnerability or shifts in family dynamics—may be essential, but how best to tailor these interventions to different caregiver-adolescent relationships is an outstanding question.

### Example of caregiver involvement in preparation and integration

In our clinical trial using psilocybin therapy for young adults with anorexia nervosa (NCT06399263), the involvement of caregivers in preparation and integration is required. We drew from a therapeutic model, EFFT, that aligns with the goal of supporting the processing and transformation of new and heightened emotions and insights that psychedelics can evoke [35]. EFFT theorizes that dysfunction in emotion processing plays a key role in the development and maintenance of mental health problems and that caregivers can be taught skills to interrupt maladaptive patterns of emotion processing (i.e., avoidance, suppression, dysregulation). Through teaching a set of emotion-coaching skills, the approach aims to increase caregivers’ capacity to attune to the emotional experience of their child. By learning to effectively mirror or validate participants’ emotional experiences, caregivers may directly support the adolescent’s self-awareness, openness, and a “rewiring” of rigid or habitual patterns of thinking and behavior, strengthening capacities for emotion regulation, cognitive flexibility, and interpersonal health [67].

**Fig. 1:**
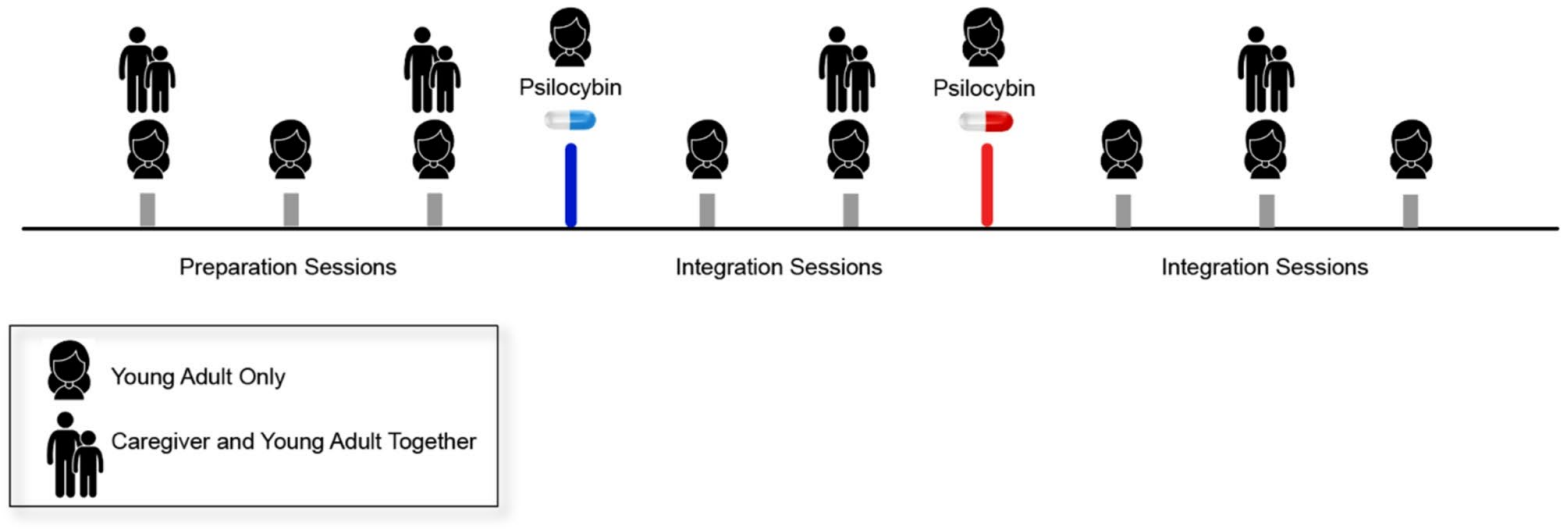
*Example of caregiver involvement in preparation and integration sessions in a clinical trial of psilocybin for young adults with anorexia nervosa****(****NCT06399263)*. *The study design includes two preparation sessions and two integration sessions where caregivers participate jointly with the young adult while also allowing time for individual therapy*. *Given the young adult population*,* the trial is designed to balance increasing autonomy with the ongoing importance of caregiver support*,* ensuring that participants receive both independent processing time and structured family involvement to enhance therapeutic outcomes and maximize safety*

Caregivers participate jointly with the young adult in several preparation and integration sessions (see Fig. 1) with overarching goals to: (1) Prepare caregivers through education about possible experiences that may arise in PAT and ways this could impact the relationship/family system, (2) clarify their role through the introduction of validation and support scripts to facilitate processing of emotion that may arise during the course of PAT, and (3) support the integration process through the practice of emotion coaching as the young adult makes meaning of their experience and explores ways to incorporate change in their life. Caregivers are coached to be maximally available following psilocybin administrations to help participants process emotions and challenging experiences that may have arisen. Likewise, participants are encouraged to communicate any needs to caregivers post-psilocybin administration.

## Conclusion

Given the growing adolescent mental health crisis and broad interest in PAT, support for PAT in adolescents is likely to increase. This review highlights the potential benefits of integrating caregivers into the varying aspects of PAT to enhance the safety and efficacy of the intervention for adolescents. Future research should begin with early-phase clinical trials that emphasize feasibility, safety, and acceptability, incorporating developmentally informed consent/assent processes and family-centered approaches. Trials should consider how best to engage caregivers while respecting adolescent needs, and should explicitly examine how developmental stage, cultural context, therapist limitations and systemic inequities may shape outcomes. Optimizing caregiver involvement requires ongoing dialogue with caregivers and individuals with lived experiences, alongside rigorous research. Ultimately, research in this area should proceed incrementally, with safeguards and multidisciplinary oversight, to ensure that adolescent participants and their caregivers are protected and empowered throughout the process.

## Data Availability

No datasets were generated or analysed during the current study.

## Abbreviations

EBT: Evidence–based treatment
PAT: Psychedelic–assisted therapy
EFFT: Emotion-focused family therapy
